# Observations on the Present State of Medicine

**Published:** 1807-12

**Authors:** 


### *To the Editors of the Medical and Physical Journal.*

Gentlemen,

WITHOUT paying any flattering encomium on T. Y's. talents, I must confess I was highly gratified by the perusal of his Letter, in No. 103 of your Journal. I think it denotes candour and liberality of sentiment; and as I perfectly agree with him on the points hinted at, I shall beg leave to suggest a few short remarks on the present state of medicine, and the respectable fraternity who have the honour nour to practise the Art. Medicine is undoubtedly arrived at a surprising pitch of perfection, and stands at the head, in my opinion, of the other sciences; for when I compare *it*, how infinitely do they fall *short*, when contrasted with the healing art. While the sciences dive into the mysteries of natural philosophy, and display the wonders of nature, medicine (collectively considered) has for its object the health of mankind. It explores that vast, that complicated machine, the human frame; and, by its powers, is often able to prolong life, by resisting the ravages of disease. It certainly confers lasting honour on the men, who have been indefatigable in their pursuits to bring it to this state of refined improvement; but, as always will, I am afraid, be the case, there are speculative characters in the world, who would, if possible, subvert the present practice of this enlightened day, and substitute the hypothetical conceits and confined systems of their own imaginations. And these unexperienced projectors in medicine, instead of delivering their theories to the world with clearness and perspicuity, promulgate their systems with such a pompous display of scholastic learning and hypothetical reasoning, that they are almost as unintelligible and obscure, as the most abstruse propositions of Euclid, to one who is not acquainted with that science.

Now, though as T. Y. justly observes, the studied formality and ridiculous garbs, that used to be the common habiliments of the profession in former days, are almost totally abolished as useless and unnecessary, yet, I think, there are other oddities *now* amongst the profession, that are almost equally disgusting, which T. Y. has not mentioned; for instance, a pompous reserve characterises one; another affects a morose supercilious behaviour; a third habituates himself to a singularity of manners, merely for the sake of being what nobody else is; a fourth puts his face into such singular contortions, even in common conversation and at the bed-side, that actually I should think must excite risibility in the beholder. Such gentlemen must recollect, that science is not concentrated in a black look, nor vast professional knowledge in a supercilious reserve. No! True learning inculcates, or ought to inculcate, affability of manners, complacency of behaviour, and a desire to inform and instruct others, rather than endeavour to concentrate every thing in self. But I would, by no means, wish to insinuate that this is *general*. The practitioners of Medicine I revere in the highest manner. These are only the foibles of a few, compared with the bulk bulk of the profession; and, therefore, it would be unjust and illiberal that a whole learned and useful profession should be blamed for the follies of a few. As to the intended reformation in the practice of medicine, it will be productive, I think, of the greatest utility; for when we reflect on the prostitution of the practice of medicine, of late years, in this metropolis, it is truly lamentable; not to mention the swarm of empirics that infest this town, how many individuals have taken upon them (without any qualifications) the responsible characters of medical practitioners, and consequently tampered with the lives of thousands—so easy was it to become a Doctor.

But now I sincerely hope, by the exertions of the Medical Reformers, that empiricism and ignorance will be banished from the profession, and science no longer abused, and effrontery and assurance countenanced. I shall encroach no further, but submit these few short remarks to the perusal of your readers.

*August* 7, 1807.

